# Promising Pharmacological Interventions for Posterior Capsule Opacification: A Review

**DOI:** 10.1002/gch2.202400181

**Published:** 2024-11-12

**Authors:** Yuxuan Liu, Xiaoming Dong, Bin Wu, Zhigang Cheng, Jinsong Zhang, Jing Wang

**Affiliations:** ^1^ AIER Cataract Institute Shenyang Liaoning Province 110000 China; ^2^ Shenyang Aier Ophthalmology Institute of Precision Medicine Shenyang Liaoning Province 110000 China; ^3^ Liaoning Aier Eye Hospital Shenyang Liaoning Province 110000 China; ^4^ Shenyang Aier Excellent Eye Hospital Shenyang Liaoning Province 110000 China; ^5^ Chaoyang Aier Eye Hospital Chaoyang Liaoning Province 122000 China; ^6^ Aier Academy of Ophthalmology Central South University No. 188, Furong South Road, Tianxin District Changsha Hunan 410004 P. R. China

**Keywords:** epithelial–mesenchymal transition, mechanism, medication, miRNA, posterior capsule opacification

## Abstract

Phacoemulsification combined with intraocular lens implantation is the primary treatment for cataract. Although this treatment strategy benefits patients with cataracts, posterior capsule opacification (PCO) remains a common complication that impairs vision and affects treatment outcomes. The pathogenesis of PCO is associated with the proliferation, migration, and fibrogenesis activity of residual lens epithelial cells, with epithelial–mesenchymal transition (EMT) serving as a key mechanism underlying the condition. Transforming growth factor‐beta 2 (TGF‐β2) is a major promotor of EMT, thereby driving PCO development. Most studies have shown that drugs and miRNAs mitigate EMT by inhibiting, clearing, or eliminating LECs. In addition, targeting EMT–related signaling pathways in TGF‐β2–stimulated LECs has garnered attention as a research focus. This review highlights potential treatments for PCO and details the mechanisms by which drugs and miRNAs counter EMT.

## Graphical abstract

This study focuses on the pathogenesis and potential treatment of posterior capsule opacification (PCO) and summarizes the mechanisms through which drugs, microRNAs, and currently investigated clinical therapies prevent epithelial‐mesenchymal transition (EMT). The findings indicate that the small mother against decapentaplegic (SMAD) pathway plays a crucial role in effectively addressing EMT. Through these research directions, it is believed that effective treatments for PCO can be developed in the near future.

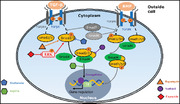

## 1 Introduction

Cataract is increasingly common among older adults and is a leading cause of blindness, affecting over 53 million people worldwide.^[^ ^1^, ^2^ ^]^ Phacoemulsification combined with intraocular lens implantation, is primary treatment for cataract, offering advantages in terms of safety, efficiency, and recovery time. However, posterior capsule opacification (PCO) is a common complication associated with this treatment strategy, leading to visual impairment and imposing mental and financial burdens.^[^ ^3^ ^]^ PCO mainly results from surgical trauma that stimulates the proliferation, epithelial–mesenchymal transition (EMT), and migration of lens epithelial cells (LECs) across the previously acellular surface of the posterior capsule or intraocular lens (IOLs).^[^ ^4^ ^]^ Neodymium‐doped yttrium aluminum garnet laser capsulotomy is commonly used to treat PCO, but it can cause complications such as corneal endothelial damage, iris hemorrhage, and uveitis.^[^ ^5^ ^]^

EMT is a central process in fibrotic PCO, although its exact pathogenesis remains unclear. This biological process involves anomalous proliferation, migration, and a notable changes in cell morphology.^[^ ^6^ ^]^ During EMT, residual LECs lose their connexin and adhesion properties,^[^ ^7^ ^]^ transforming into mesenchymal cells.^[^ ^8^ ^]^ These cells differentiate abnormally into fiber cells, known as Soemmering's ring and Elschnig's pearls,^[^ ^3^ ^]^ and migrate to the posterior capsule, ultimately causing PCO.^[^ ^9^ ^]^ EMT models of LECs, based on relevant growth factors, can be used to study fibrotic PCO.

In this review, we summarize the potential therapeutic mechanisms of drugs and miRNAs on EMT in in vitro models and explore clinical treatments currently under investigation.

## 2 Induction of Growth Factors in EMT

Cataract surgery can disrupt the blood–aqueous barrier, leading to the release of transforming growth factor‐beta(TGF‐β), interleukins, interferons, and other cytokines.^[^ ^10^ ^]^ Studies have shown that TGF‐β triggers pathological changes in LECs similar to those observed in human anterior subcapsular cataract.^[^ ^11^ ^]^ TGF‐β2, the main isoform of TGF‐β in aqueous humor,^[^ ^12^ ^]^ has a 10‐fold greater potency to induce opacification than TGF‐β1.^[^ ^13^ ^]^

Small mother against decapentaplegic (SMAD) proteins play various roles and can be classified into inhibitory SMADs (SMAD6 and SMAD7), receptor‐activated (R)‐SMADs (SMAD2/3 and SMAD1/5/8), and SMAD4, which serves as a partner for activated R‐SMADs.^[^ ^14^, ^15^ ^]^ In response to TGF‐β, SMAD2/3 are phosphorylated by the TGF‐βR1 receptor, whereas SMAD1/5/8 respond to bone morphogenetic proteins as well as growth and differentiation factors and are phosphorylated by the TGF‐βR1 receptor.

Furthermore, hepatocyte growth factor (HGF) is highly expressed throughout all stages of human capsular bag culture in a protein‐free medium^[^ ^16^ ^]^ and promotes LEC proliferation, suggesting that it may contribute to PCO development.^[^ ^17^ ^]^ HGF drives EMT by binding to its receptor, c‐MET, and a protein with tyrosine kinase activity that activates downstream signaling pathways, including the phosphatidylinositol 3‐kinase (PI3K)/AKT serine/threonine kinases/mammalian target of rapamycin (mTOR), Ras/mitogen‐activated protein kinase, and Janus tyrosine kinase (JAK)/signal transducer and activator of transcription (STAT) signaling pathways; these pathways regulate cell growth, migration, and invasion.^[^ ^18^, ^19^, ^20^ ^]^

## 3 Effects of Drugs on EMT in In Vitro Models

### 3.1 Rapamycin

Rapamycin, a natural antibiotic isolated from *Streptomyces hygroscopicus*,^[^ ^21^ ^]^ functions as a potent immunosuppressant, an anticancer agent with low toxicity and above‐average efficiency, and a specific mTOR inhibitor.^[^ ^21^, ^22^, ^23^ ^]^ It has been shown to inhibit the LEC proliferation in an in vitro rabbit model^[^ ^24^ ^]^ and enhance apoptosis in human LECs (HLECs; HLE‐B3 cells).^[^ ^25^ ^]^ The upstream regulators of the mTOR signaling pathway include PI3K/AKT, Ras, and AMP‐activated protein kinase (AMPK).^[^ ^26^ ^]^ PI3K plays a crucial role in the regulation of mTOR activity by enhancing AKT phosphorylation.^[^ ^27^ ^]^ In 2014, Tian et al.^[^ ^28^ ^]^ showed that rapamycin not only strongly suppressed the proliferation of HLECs but also induced their apoptosis in a dose‐dependent manner under HGF stimulation. Rapamycin also induces HLEC apoptosis by inhibiting HGF‐induced phosphorylation of AKT/mTOR, ERK, and JAK2/STAT3 signaling molecules, thereby attenuating EMT progression.^[^ ^28^ ^]^

However, a recent study reported that rapamycin promotes EMT by activating autophagy, a highly conserved process that helps maintain cellular homeostasis by degrading and recyclerecycling proteins and organelles.^[^ ^29^ ^]^ In 2021, Sun et al. found that rapamycin amplified TGF‐β2‐induced EMT in rabbit LECs and increased p‐SMAD2 and p‐SMAD3 levels compared with TGF‐β2 treatment alone.^[^ ^30^ ^]^ Furthermore, rapamycin activated autophagy, boosted EMT‐related marker expression, and reversed the inhibitory effects of blue light (455 nm) on TGF‐β2 induced EMT.^[^ ^31^ ^]^ The relationship between rapamycin and EMT remains controversial, as crosstalk exists between EMT and autophagy or apoptosis signaling pathways. The effects of rapamycin on EMT vary depending on the cytokines involved. Thus, understanding how different cytokines interact with rapamycin may offer insights into preventing PCO in the future.

### 3.2 Aldose Reductase Inhibitors

Aldose reductase catalyzes the conversion of glucose to sorbitol in the first step of the polyol pathway. A previous study reported that aldose reductase inhibitors attenuate the proliferation of LECs and the expression of EMT biomarkers, such as alpha‐SMA, beta‐crystallin, and ICAM‐1; however, the precise role of aldose reductase in PCO remains unclear.^[^ ^32^ ^]^ A strong link between aldose reductase and PCO has been established, with aldose reductase overexpression driving the expression of EMT and PCO biomarkers.^[^ ^33^ ^]^

Chang et al.^[^ ^34^ ^]^ found that sorbinil‐mediated aldose reductase inhibition or siRNA‐mediated aldose reductase gene knockdown under TGF‐β induced conditions not only suppressed SMADs phosphorylation and matrix metalloproteinase activation but also reduced EMT marker expression. In 2018, Zukin et al.^[^ ^35^ ^]^ showed that aldose reductase inhibition, both pharmacologically (sorbinil) and via gene knockdown, attenuated EMT in vivo in a mouse cataract surgery model. Whether used in vivo or in vitro models, aldose reductase inhibitors exhibit strong inhibitory effects on EMT. Aldose reductase upregulation has been linked to increased pERK1/2 levels in transgenic mice,^[^ ^36^ ^]^ with aldose reductase inhibitors reversing this upregulation in diabetic rats.^[^ ^37^ ^]^ TGF‐β2 also activates the RAS/MEK/ERK MAP kinase cascade, independent of SMAD4, to modulate matrix contraction.^[^ ^38^ ^]^ Further research is needed to clarify whether aldose reductase inhibitors affect TGF‐β2–induced EMT via SMADs.

### 3.3 Metformin

Metformin is an oral drug widely used to treat hyperglycemia, especially in patients with type 2 diabetes mellitus. Preclinical studies have shown that metformin can inhibit cancer cell growth.^[^ ^39^, ^40^ ^]^ Most researchers attribute its anticancer activity to the activation of AMPK and/or the reduction of serum insulin levels.^[^ ^41^, ^42^, ^43^ ^]^ In addition, activated AMPK directly phosphorylates and activates tuberous sclerosis complex 2, inhibiting the mTORC1 signaling pathway, which in turn suppresses mRNA translation and ultimately cell growth and proliferation.^[^ ^44^ ^]^

In 2018, Wang et al.^[^ ^44^ ^]^ found that metformin inhibits multiple myeloma cell lines by inducing autophagy and disrupting the cell cycle at the G0/G1 phase. Moreover, metformin not only activates AMPK phosphorylation but also reduces mTORC phosphorylation. Notably, Patnaik et al.^[^ ^45^ ^]^ reported that diabetic patients taking metformin after phacoemulsification had a lower risk of PCO and subsequent YAG capsulotomy. Furthermore, in the presence of TGF‐β2, metformin has been shown to suppress SMAD2/3 phosphorylation and nuclear translocation by activating AMPK phosphorylation.^[^ ^46^ ^]^ Conversely, compound C, an AMPK inhibitor, reverses metformin's inhibitory effects on the EMT of HLECs and the TGF‐β/SMAD2/3 signaling pathway.^[^ ^46^ ^]^ These studies highlight the potential of metformin as a novel therapeutic drug for EMT. Although AMPK phosphorylation plays a critical role in metformin's effects on EMT, the downstream factors of AMPK remain unclear. Identifying these factors, especially those affecting the SMAD pathway, will be a key focus for future research.

### 3.4 Capsaicin

Capsaicin, a pungent alkaloid found in red pepper (*Capsicum annuum*), has notable anti‐inflammatory and antioxidative properties.^[^ ^47^, ^48^ ^]^ A close relationship exists between capsaicin and transient receptor potential (TRP) cation channel proteins. TRPs are a nonselective cation superfamily involved in initial physical responses to various stimuli.^[^ ^49^ ^]^ Two members of the vanilloid subfamily of TRPs, namely, TRP vanilloid 1 (TRPV1) and TRPV4, are expressed in mouse lenses.^[^ ^50^ ^]^ TRVP1, activated by heat, vanilloids, acidic pH, and capsaicin,^[^ ^51^, ^52^ ^]^ modulates lens volume and hydrostatic pressure while maintaining the hydrostatic pressure gradient.^[^ ^53^ ^]^

In 2021, Sugiyama et al.^[^ ^54^ ^]^ used immortalized HLECs and lens epithelial explants to perform an injury‐induced EMT assay in vivo. They found that capsaicin not only inhibited LEC proliferation but also suppressed EMT in primary explant LECs following pretreatment. Notably, capsaicin eye drops inhibited EMT in vivo. Furthermore, capsaicin regulates TGF‐β2‐induced SMAD2/3 phosphorylation by suppressing the EGFR/ERK pathway. These findings highlight the potential of capsaicin as a therapeutic drug for PCO.

In another study, de Jong et al.^[^ ^55^ ^]^ found that activated TRPV1 suppressed EGFR‐induced intestinal epithelial cell proliferation by activating calpain, which in turn activated protein tyrosine phosphatase 1B (PTP1B). In 2024, Huang et al.^[^ ^56^ ^]^ demonstrated that TRPV1 activation through mannitol and distilled water‐induced hyperosmotic stress, activated autophagy through the Ca^2+^‐dependent AMPK/mTOR pathway, affecting cellular migration, and inhibiting PCO progression. Therefore, TRVP1 appears to be a key factor in capsaicin's ability to prevent PCO, although further research is needed to explore the role played by PTP1B downstream of TRVP1 in blocking the EGRF pathway. This will be a promising direction for further studies on capsaicin.

### 3.5 Resveratrol

Resveratrol, a natural polyphenolic phytoalexin found in red wine and other foods, has shown several potential health benefits.^[^ ^57^ ^]^ It can hinder liver fibrosis development by reducing fibrosis‐related gene expression^[^ ^58^ ^]^ as well as reduce inflammation and fibrosis in patients with Crohn's disease.^[^ ^59^ ^]^ In 2015, Ishikawa et al.^[^ ^60^ ^]^ revealed that resveratrol inhibits TGF‐β2‐induced EMT in human retinal pigment epithelial cells by deacetylating SMAD4. Smith et al.^[^ ^61^ ^]^ confirmed that resveratrol markedly impedes LEC migration and suppresses TGF‐β2‐induced EMT biomarker expression as well as TGF‐β2‐induced capsular wrinkling and fibrotic disease‐associated gene expression. However, further research is needed to determine the feasibility and efficacy of resveratrol as a therapeutic agent.

### 3.6 Aspirin

Aspirin (acetylsalicylic acid), one of the oldest and most widely used medications, is commonly employed to treat pain, fever, and inflammation.^[^ ^62^, ^63^ ^]^ It modulates the acetylation of lysine residues in cellular and extracellular proteins^[^ ^64^, ^65^ ^]^ and can prevent cataract formation by inhibiting lens protein acetylation.^[^ ^66^ ^]^ EMT can also be inhibited through the histone acetylation of arginine or lysine residues.^[^ ^67^, ^68^ ^]^ In 2020, Nam et al.^[^ ^69^ ^]^ assessed the effects of aspirin on TGF‐β2‐induced EMT in HLECs, LECs in human lens capsular bags, and lensectomized mice. They found that aspirin markedly suppressed EMT biomarker expression and reduced posterior capsule wrinkling. The molecular mechanism, as demonstrated through chromatin immunoprecipitation assays, involves aspirin blocking TGF‐β2‐induced acetylation of K56 and K122 in the H3 histone at the promoter regions of the actin alpha 2 and collagen type I alpha 1 genes. This disrupts SMAD binding to EMT gene promoter regions.^[^ ^69^ ^]^ Furthermore, cyclooxygenase‐2 (COX‐2), a target of aspirin,^[^ ^70^ ^]^ has been associated with TGF‐β1‐induced EMT in bladder transitional cell carcinoma and was shown to mediate EMT in human breast cancer cells via the TGFβ/SMAD3 pathway.^[^ ^71^ ^]^ In 2022, Feiyue et al.^[^ ^72^ ^]^ found that aspirin‐soaked intraocular lenses could suppress the migration of SRA01/04 cells by continuously releasing aspirin in an in vitro lens capsule model. This finding supports aspirin's ability to downregulate EMT, although the exact mechanism remains unclear. Thus, further research on whether aspirin inhibits EMT by targeting COX‐2 to regulate SMAD3 is a critical area for future investigation (**Table** **1**).

**Table 1:** Summary of the mechanisms by which various drugs inhibit the EMT process in models.

Drugs	Main mechanism	Cells	Model	Refs.
Rapamycin	‐Induces the apoptosis by inhibiting HGF‐induced AKT/mTOR, ERK, and JAK2/STAT3 phosphorylation ‐Augments TGF‐β2‐induced EMT in LECs Boosts p‐SMAD2 and p‐SMAD3 ‐Activates autophagy Strengthens expression of EMT related marker Reversed the blue light inhibition efficacy for EMT	HLECs	Vitro	[28]
		Primary rabbit LECs	Vitro	[30]
		HLE‐B3	Vitro	[31]
Aldose reductase inhibitor (Sorbinil)	‐Attenuates TGF‐β2‐induced SMAD2/3 activation Interrupts AR‐SMAD2 and SMAD2‐SMAD4 interaction Decreases TGF‐β2‐induced MMP‐2 and MMP‐9 Down‐regulating the expression of EMT markers (a‐SMA and vimentin)	HLE‐B3 Mice primary LECs	Vitro	[34]
Metformin	‐Suppresses TGF‐β2‐induced SMAD2/3 phosphorylation and nuclear translocation Activates AMPK phosphorylation Increases the expression of E E‐cadherin and decreases the expression of collagen I, vimentin, α‐SMA, and FN1	HLE‐B3	Vitro	[46]
Capsaicin	‐Modulates TGFβ2‐induced SMAD2/3 activation by inhibiting EGFR/ERK pathway Reduces expression of EMT‐related genes(a‐SMA, COL1A1, and CTGF) Binds TRVP1 to inhibit EGFR/ERK pathway	HLECs Rat lens epithelial explants Mice	Vitro Vitro Vivo	[54]
Resveratrol	‐Does not prevent TGFβ2‐induced SMAD2/3 nuclear translocation Prevents the expression of a‐SMA and FN Suppresses the expression of MMP2	FHL124 Human capsular bag Central anterior lens epithelia	Vitro Vitro Vitro	[61]
Aspirin	‐Inhabits expression of EMT‐related genes (α‐SMA and αB‐crystallin) Attenuate EMT through acetyl moiety Blocks the TGF‐β2‐induced acetylation of K56 and K122 in the H3 histone at the promoter regions of actin alpha 2 (ACTA2), affecting the combination between SMAD4 and promoter region of EMT genes.	FHL124	Vitro	[69]

## 4 Biological Functions of microRNAs (miRNAs) in EMT‐Related Signaling Pathways

miRNAs are vital modulators of numerous physiological processes, including gene expression regulation through binding to the 3′‐untranslated region (3′‐ UTR) of mRNAs and triggering mRNA degradation or translation repression.^[^ ^73^ ^]^

### 4.1 miRNAs Related to the TGF‐β/SMAD Signaling Pathway

SMAD proteins function as intracellular mediators that transduce extracellular signals from TGF‐β ligands to the nucleus, where they activate downstream TGF‐β gene transcription.^[^ ^74^, ^75^ ^]^ Among them, SMAD4 is critical to the SMAD pathway as it binds to phosphorylated SMAD2/3 (p‐SMAD2/3) to form a heteromeric complex with the TGF‐β ligand, thereby regulating the canonical TGF‐β signaling pathway.^[^ ^76^ ^]^

Wang et al.^[^ ^77^ ^]^ conducted a microRNA array study and found that miR‐204‐5p expression was significantly downregulated in the PCO group (PCO tissue) compared with that in the normal group (samples of normally attached LECs obtained from an eye bank). Furthermore, miR‐204‐5p inhibited SMAD4 expression but not p‐SMAD2/3 expression. Notably, the overexpression of miR‐204‐5p markedly inhibited TGF‐β2‐induced EMT.^[^ ^77^ ^]^ In another study, lncRNA‐MALAT1 downregulated miR‐204‐5p, thereby regulating TGF‐β signaling pathway inhibition and preventing EMT.^[^ ^78^ ^]^

Numerous studies have shown that miR‑486‐5p inhibits cancer, including prostate and colorectal cancers, through targeting specific genes.^[^ ^79^, ^80^ ^]^ Liu et al.^[^ ^81^ ^]^ revealed that miR‐486‐5p directly targets the 3′‐UTR of *SMAD2*, attenuating TGF‐β2‐induced SMAD2/3 phosphorylation in LECs (SRA01/04). However, the target of miR‐486‐5p remains controversial. Wang and Zheng^[^ ^82^ ^]^ showed that lncRNA‐NEAT1 interacts with miR‐486‐5p and regulates the TGF‐β2/SMAD signaling pathway by diminishing the inhibitory effect of miR‐486‐5p on SMAD4, thereby promoting proliferation, migration, invasion, and EMT in LECs. Therefore, whether miR‐486‐5p inhibits complex formation between SMAD4 and activated SMAD2/3 by targeting SMAD4 and modulating the TGF‐β2/SMAD pathway is a critical area warranting further investigation.

miR‐34a has been shown to hinder tumorigenesis in various cancers, including gastric, prostate, and breast cancers.^[^ ^83^, ^84^, ^85^ ^]^ Multiple studies have shown that the Notch signaling pathway is associated with tumor cell proliferation, metastasis, EMT, and apoptosis.^[^ ^86^, ^87^, ^88^, ^89^ ^]^ The Jagged1/Notch pathway, which is downstream of the canonical TGF‐β2/SMAD signaling pathway, is activated by TGF‐β2.^[^ ^90^ ^]^ This was confirmed by Han et al.,^[^ ^91^ ^]^ who found that miR‐34a targets Notch1, thereby suppressing TGF‐β2‐induced EMT in LECs. Notably, in TGF‐β2‐induced LECs, lncRNA‐NEAT1 exerts a negative regulatory effect on miR‐34a, competing with miR‐34a to alleviate its inhibitory impact on the target gene *Snail1*.^[^ ^92^ ^]^ The Snail1 protein, a transcription factor, is closely associated with EMT in LECs^[^ ^93^ ^]^ and is regulated in the TGF‐β pathway via a heterooligomeric complex formed via the combination of activated SMAD2/3 and SMAD4.^[^ ^94^ ^]^ In summary, microRNAs associated with the TGF‐β/SMAD pathway typically target crucial pathway components, thereby influencing EMT in LECs. However, research in this area remains limited, necessitating ongoing validation through in vivo and in vitro studies.

### 4.2 miRNAs Related to Other Signaling Pathways

The PI3K/AKT signaling pathway, a SMAD‐independent pathway, plays a crucial role in the TGF‐β2‐induced development of LECs, influencing cell proliferation, migration, and EMT.^[^ ^95^, ^96^ ^]^ Research has indicated that miR‐26b expression is significantly reduced in EMT models.^[^ ^97^ ^]^ Furthermore, overexpressing miR‐26b not only suppresses proliferation, migration, and EMT in TGF‐β2‐induced LECs but also inhibits both PI3K and AKT phosphorylation.^[^ ^97^ ^]^

Histone deacetylases (HDACs) are essential enzymes that regulate chromatin condensation and gene transcription through the deacetylation of lysine residues in histones.^[^ ^98^ ^]^ HDAC6, a prominent cytoplasmic deacetylase, uniquely binds to cytoplasmic substrates to induce signaling pathways involved in embryonic development, tumorigenesis, and neurodegenerative diseases, among others.^[^ ^99^, ^100^ ^]^ In addition, HDAC6 is implicated in renal and pulmonary fibrosis.^[^ ^101^ ^]^ Several studies have demonstrated that miR‐22‐3p acts as a tumor suppressor, inhibiting various cancers, such as hepatocellular carcinoma, ovarian cancer, colon cancer, and gastric cancer, by reducing cell proliferation and tumorigenicity.^[^ ^102^, ^103^, ^104^, ^105^ ^]^ *HDAC6* has been identified as a target of miR‐22‐3p.^[^ ^106^ ^]^ Moreover, α‐tubulin, an essential cytoskeletal component, serves as a substrate for HDAC6.^[^ ^107^ ^]^ Wang et al.^[^ ^108^ ^]^ found that miR‐22‐3p inhibits LEC proliferation, migration, and TGF‐β2‐induced EMT by downregulating HDAC6 activity and increasing α‐tubulin acetylation (**Table** **2**).

**Table 2:** Summary of the mechanisms by which various miRNAs inhibit the EMT process in in vitro models.

miRNAs	Target	Mechanism	Cells	Refs
miR‐204‐5p	SMAD4	‐Down‐regulates the expression of SMAD4 Affects SMAD2/3 phosphorylation	Human PCO tissues	[77]
miR‑486‐5p	SMAD2 SMAD4	‐Down‐regulates the expression of SMAD2 Suppresses SMAD2/3 phosphorylation ‐Down‐regulates the expression of SMAD4 Affects the combination of SMAD4 and activated SMAD2/3	SRA01/04	[81]
			SRA01/04	[82]
miR‐34a	Notch1 Snail1	‐Regulates Jagged1 and Notch1 negatively Suppresses TGFβ2‐induced EMT by hindering Notch1 ‐Down‐regulates the expression of Snail1	Human anterior capsule samples	[91]
			SRA01/04	[92]
miR‐26b	/	‐Inhibits activation of the PI3K/Akt pathway induced by TGFβ‐2	HLE‐B3	[97]
miR‐22‐3p	HDAC6	‐Prevents lens fibrotic progression by targeting HDAC6 thereby promoting α‐tubulin acetylation	Human lens epithelial explants	[108]

## 5 Effects of PCO Clinical Management Strategies under Investigation

Current clinical approaches for managing PCO include 1) redesigned artificial IOLs implanted in the capsular bag to prevent PCO,^[^ ^109^ ^]^ 2) laser capsulotomy, and 3) ocular drug delivery.^[^ ^110^ ^]^ Although laser capsulotomy offers convenience, it can lead to complications, such as intraocular inflammation, IOL displacement, and macular edema.^[^ ^111^, ^112^ ^]^ Research on ocular drug delivery to the anterior segment of the eye found that this approach overcomes ocular barriers, controls drug release, attenuates toxicity, and mediates bioavailability.^[^ ^110^, ^113^, ^114^, ^115^ ^]^ The development of drug delivery systems (DDSs) for PCO is rapidly advancing, with a focus on drug‐loaded IOLs, nanocarriers, hydrogel composites, and implants.^[^ ^116^ ^]^ Two common methods for drug‐loaded IOLs include 1) loading the optical surface and rim, and 2) applying nanocarriers and free drugs to the haptics of the IOL.^[^ ^116^ ^]^ Below, we summarize some drugs incorporated into DDS to prevent PCO.

### 5.1 Drug‐Loaded IOL

Nanomaterials are widely used in PCO treatment. In 2021, Gautam et al.^[^ ^117^ ^]^ created a sorbinil‐eluting nanogel attached to mouse‐sized IOL‐like substrates, which were implanted in the mouse lens capsule after mock cataract surgery, effectively preventing PCO in mice. Ye et al.^[^ ^118^ ^]^ developed Rapa@Ti3C2 (rapamycin‐loaded Ti3C2 nanosheets) deposited on IOLs with surface activation, allowing drug release upon near‐infrared (NIR) light stimulation and preventing PCO for 4 weeks without damaging ocular structures in female chinchilla rabbits. In addition, modifying the IOL's surface with polyethyleneimine (PEI) overnight generates a positive charge. Zhu et al.^[^ ^119^ ^]^ demonstrated that DOX@Exos‐IOL (a PEI‐modified IOL immersed in a doxorubicin‐loaded exosome suspension) reduced residual LEC activity after phacoemulsification in rabbit models through sustained drug release.

Physical coating and chemical grafting are also effective methods for drug loading on IOLs. Zhang et al.^[^ ^120^, ^121^ ^]^ fabricated drug‐eluting IOLs for bromfenac and indomethacin using ultrasonic spray technology and degradable poly(lactic‐co‐glycolic acid) as the drug carrier. Both bromfenac and indomethacin are nonsteroidal anti‐inflammatory drugs that inhibit COX‐2.^[^ ^122^, ^123^ ^]^ Bromfenac suppresses the MEK/ERK signaling pathway, hinders glycogen synthase kinase‐3β (GSK‐3β), and blocks downstream factors, such as Snail, a target of EMT‐related genes in HLE‐B3.^[^ ^120^ ^]^

Indomethacin downregulates COX‐2 and EMT markers, activates autophagy in HLE‐B3, and reduces p62 levels to modulate Snail.^[^ ^121^ ^]^ In rabbit PCO models, bromfenac/indomethacin‐eluting IOLs demonstrated prominent anti‐inflammatory and anti‐PCO effects.

### 5.2 Non‐IOL Dosage Forms

In 2016, a capsular tension ring (CTR) was developed through a two‐step process using 2‐hydroxyethyl methacrylate and methyl methacrylate as raw materials to deliver docetaxel (DTX).

Lei et al.^[^ ^124^ ^]^ showed that DTX‐CTR effectively inhibited PCO in rabbit models after surgery without damaging normal tissues. In vitro, DTX‐CRT exhibited superior bending strength to support the capsular bag and provided sustained drug release for up to 6 weeks.

Nanoparticle–hydrogel composites, consisting of nanoparticles and gel solutions, can be administered through periocular or intracameral injection. Yan et al.^[^ ^125^ ^]^ developed a temperature‐sensitive DDS (GenNLC‐DEX‐MOX hydrogel) containing dexamethasone (DEX), moxifloxacin (MOX), and a genistein nanostructured lipid carrier (GenNLC). This hydrogel exhibits diverse drug‐release kinetics and effectively inhibits the proliferation, migration, and EMT of LECs.

## 6 Conclusion and Future Perspectives

Research has identified several key pathways that can be targeted to suppress EMT, such as the TGF‐β/SMAD and PI3K/AKT signaling pathways. This review highlights the potential of miRNA‐based treatments by describing miRNAs that target the key components of these pathways. In addition, various pharmacological agents, such as sorbinil, metformin, capsaicin, resveratrol, and aspirin, were found to prevent EMT and PCO by targeting the SMAD pathway. Many miRNAs involved in inhibiting EMT in LECs rely on the SMAD pathway, particularly by blocking nuclear translocation of activated SMAD, which in turn suppresses the expression of EMT‐related proteins. Thus, the SMAD pathway remains a vital focal point in EMT suppression.

The effectiveness of drug‐loaded IOLs is closely associated with EMT inhibition mechanisms. Both the aldose reductase inhibitor sorbinil and rapamycin have shown promise in attenuating EMT in vitro. Sorbinil‐eluting nanogels, which are covalently linked to IOL surfaces, overcome drug delivery challenges, such as limited drug concentrations and corneal barriers, effectively inhibiting the mesenchymal transition of LECs. Rapamycin, known for blocking AKT/mTOR, ERK, and JAK2/STAT3 phosphorylation to induce autophagy, has been successfully applied in eluting stents during clinical trials.^[^ ^126^, ^127^ ^]^ Rapa@Ti3C2‐IOL, created using a two‐step spin‐coating method, boasts superior photothermal conversion and can be triggered by NIR, preventing PCO.^[^ ^118^ ^]^ The transcription factor Snail plays a crucial role in EMT occurrence and progression in cancer.^[^ ^128^ ^]^ Meanwhile, GSK‐3β binds to Snail to maintain the epithelial phenotype.^[^ ^129^ ^]^ Snail is also implicated in the inhibition of EMT by bromfenac/indomethacin‐eluting IOLs. Studies have shown that autophagy activation helps reduce high glucose‐induced EMT by suppressing the accumulation of p62 and Snail,^[^ ^130^ ^]^ a mechanism similar to the activation of autophagy and inhibition of EMT by indomethacin. In addition, bromfenac inhibits ERK phosphorylation, suppresses GSK‐3β phosphorylation and inactivation, and binds Snail, thereby downregulating EMT‐related gene expression. Notably, this inhibition occurs without affecting SMAD2/3 phosphorylation, indicating that Snail may act as a pivotal factor in preventing EMT via SMAD‐independent pathways.

Most studies conducted on PCO have used cells derived from human or animal lenses, offering both benefits and limitations. These cell models help advance our understanding of PCO development as they maintain their primitive epithelial phenotype and responsiveness to external stimuli. In addition, they allow researchers to observe changes in cell morphology, proliferation, and differentiation, providing insights into the cellular dynamics of PCO.^[^ ^131^ ^]^ However, these in vitro‐cultured cells have certain limitations. For example, cultured LECs often differ from native epithelial cells and the residual cells in the lens capsule after surgery. Furthermore, PCO manifests in two forms, i.e., regenerative and fibrous, as a result of different types of epithelial cells. Fibrous PCO, characterized by excessive proliferation, migration, invasion, and EMT of residual LECs,^[^ ^132^ ^]^ is commonly studied using TGF‐β2‐induced EMT models in vitro. However, such models do not fully replicate the complexity of PCO development in vivo, where biological conditions are more multifaceted. Although in vitro studies are informative, they cannot completely capture the lens capsule's natural proliferation, migration, and contraction processes.^[^ ^133^ ^]^ Thus, validating findings through in vivo experiments is crucial after completing in vitro assessments.

Although drugs and miRNAs have shown promise in preventing EMT in vitro, their in vivo efficacy and safety remain underexplored. A key challenge is the efficient and safe delivery of these therapies to the lens capsule, ideally with minimal toxicity to surrounding tissues, while specifically targeting LECs.

This review illustrated the potential of drug‐loaded IOLs and non‐IOL dosage forms, highlighting both advantages and limitations. Their advantages include 1) reduced toxicity to ocular tissues; 2) sustained, slow release of treatments for PCO; and 3) versatile drug‐release kinetics. However, their limitations include 1) reliance on animal models, primarily rabbits, and HLECs; 2) complex production processes; and 3) uncertain long‐term safety of the delivery systems. Future research on PCO should focus on the following key areas: 1) optimization of in vitro and in vivo models, 2) drug pharmacology using in vivo models, 3) long‐term safety of DDSs in ocular tissues, and 4) specific targets and mechanisms of miRNAs involved in EMT‐related signaling pathways. Through these research directions, we believe that effective treatments for PCO can be developed in the near future.

## Conflict of Interest

The authors declare no conflict of interest.

## Author Contributions

Y.L., X.D., and B.W. cooperated in the search of databases. Y.L., X.D., Z.C., and J.W. carried out the drafting of the manuscript and designed the structure of the manuscript. J.Z. has provided suggestions for revising the manuscript. All authors read and approved the final manuscript.
